# Cigarette Smoke May Increase Microbial Virulence

**DOI:** 10.1289/ehp.121-a75

**Published:** 2013-03-01

**Authors:** David C. Holzman

**Affiliations:** David C. Holzman writes on science, medicine, energy, economics, and cars from Lexington and Wellfleet, MA. His work has appeared in *Smithsonian*, *The Atlantic Monthly*, and the *Journal of the National Cancer Institute*.

Cigarette smoke has long been known to aggravate respiratory infections. A new study expands the potential health effects to a new front, showing that acute *in vitro* exposure of *Staphylococcus aureus* to cigarette smoke promoted biofilm formation and adhesion to human cells.^1^ The active agents behind this boost in activity appeared to be reactive oxygen species (such as hydrogen peroxide), which are linked not just to cigarette smoke but also vehicular exhaust and biomass smoke. The research was conducted by Ritwij Kulkarni, a postdoctoral researcher in the laboratory of Adam Ratner at the Columbia University Department of Pediatrics.

The new research “may in part explain the strong association between cigarette smoke exposure and respiratory tract infections,” says Janet Lee, an associate professor of medicine at the University of Pittsburgh Medical Center, who was not a collaborator on the study. Earlier research had linked cigarette smoke with biofilm formation,^2^ but this is the first to examine potential underlying mechanisms, she says.

In the current study, the researchers exposed multiple strains of cultured *S. aureus* to various concentrations of cigarette smoke by bubbling the smoke into the growth medium. Biofilm formation increased in exposed *S. aureus* in a dose-dependent fashion. Exposed bacteria also showed increased binding to human fibronectin and lung epithelial cells, compared with controls. Fibronectin is a cell-surface protein that aids in cellular adhesion, among other roles. Bacteria may bind to fibronectin in order to invade human cells.

Exposure to hydrogen peroxide alone also induced biofilm production, whereas spiking cigarette smoke with the antioxidant *N*-acetyl cysteine interrupted its ability to induce biofilm formation. These observations suggest that an oxidant-dependent pathway is triggered by smoke, leading to enhanced biofilm formation.

The paper is part of a new but rapidly expanding literature. Noam A. Cohen, an assistant professor in the Department of Otorhinolaryngology, Head and Neck Surgery at the University of Pennsylvania, has investigated cigarette smoke’s effect on multiple species of bacteria at once. The bacteria were isolated from sinonasal passages of patients with chronic sinusitis, both smokers and nonsmokers. Cigarette smoke exposure was associated with increased biofilm formation among all species of bacteria, but more so among those isolated from smokers than in those from nonsmokers.^2^

**Figure:**
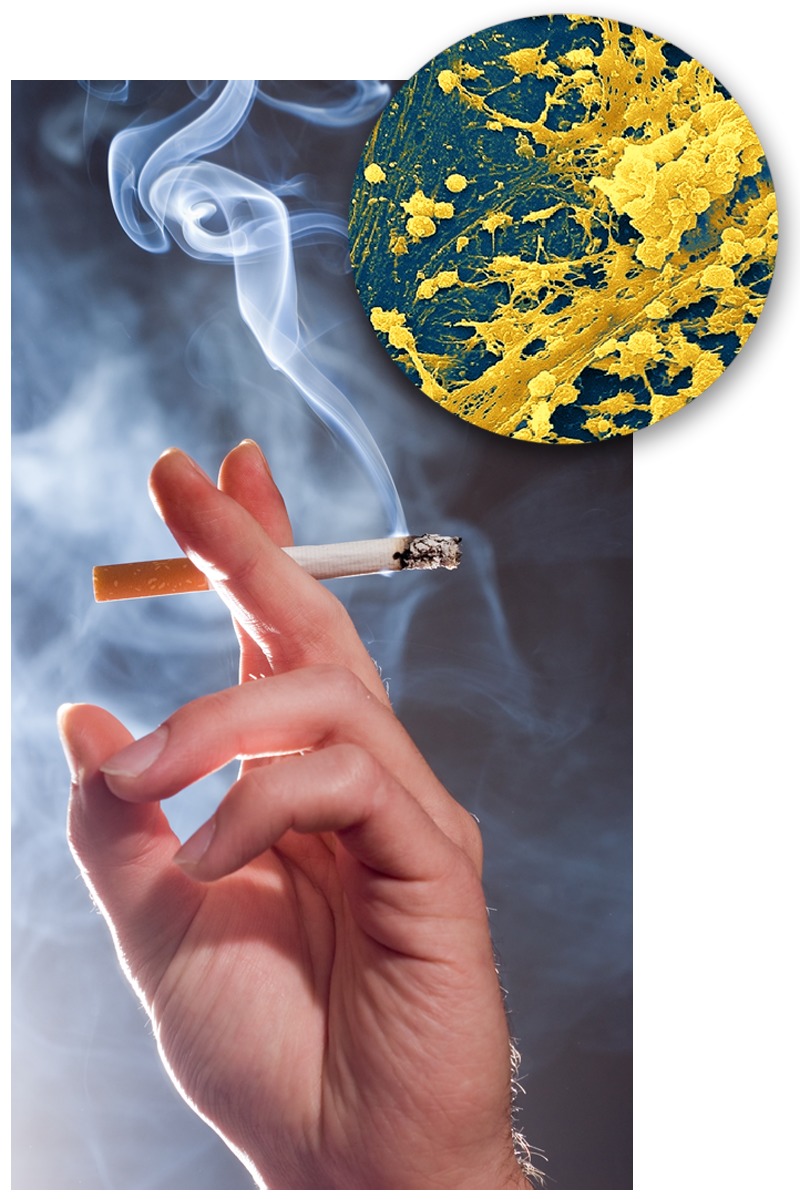
Reactive oxygen species in cigarette smoke may promote biofilm formation by Staphylococcus aureus (inset), a common respiratory pathogen. Hand: © Shutterstock.com; S. aureus inset: © Science Source

Cohen praises the Columbia study and says the new work suggests tobacco smoke may “convert the bacteria to a more aggressive form.” But more studies are needed to confirm the findings and determine whether enhanced biofilm formation translates into greater microbial pathogenicity or persistence; Cohen’s own data suggest that cessation of smoke exposure reverses the boost in biofilm formation.^2^ Furthermore, the use of cigarette smoke extract for *in vitro* studies has not been standardized.

The relevance of all these findings to human environmental health is broad, says Lee, a clinician with an interest in how cigarette smoke alters immune cell populations. “Cigarette smoke has been linked to a number of diseases, such as oral gingivitis, chronic rhinosinusitis, and chronic lung diseases,” she says, and epidemiologic evidence suggests that exposure to secondhand smoke increases the risk of *S. aureus* colonization in children.^3^ Other new findings show that cigarette smoke enhances biofilm production of *Pseudomonas aeruginosa*^4^ and *Streptococcus pneumoniae*,^5^ two very important respiratory pathogens. “But the pathogenic mechanisms are still not well understood,” Lee says, calling the new research “an important step in identifying which genes are involved.”

All this work may have particular relevance for patients with cardiovascular disease. “This could definitely be part of the comorbidities they are seeing with these patients that aren’t well explained by the cardiovascular diseases,” says Jeff G. Leid, an associate professor at of the Center for Microbial Genetics and Genomics at Northern Arizona University.

Cohen adds that cigarette smoke exposures go well beyond the respiratory tree. He explains, “Many of the products of tobacco get into the blood stream and can thus affect bacteria in distant locations”—in the joints, heart, and gastrointestinal tract, for example. Concludes Ratner: “Our environmental exposures don’t just affect us; they affect our bacteria.” Whether this helps us or hurts us, he says, we need to take it into consideration when investigating the health effects of environmental exposures.
